# Altered Modular Organization of Structural Cortical Networks in Children with Autism

**DOI:** 10.1371/journal.pone.0063131

**Published:** 2013-05-10

**Authors:** Feng Shi, Li Wang, Ziwen Peng, Chong-Yaw Wee, Dinggang Shen

**Affiliations:** 1 Department of Radiology and BRIC, University of North Carolina at Chapel Hill, Chapel Hill, North Carolina, United States of America; 2 Department of Psychology, South China Normal University, Guangdong, China; 3 Department of Brain and Cognitive Engineering, Korea University, Seoul, Korea; Indiana University, United States of America

## Abstract

Autism is a complex developmental disability that characterized by deficits in social interaction, language skills, repetitive stereotyped behaviors and restricted interests. Although great heterogeneity exists, previous findings suggest that autism has atypical brain connectivity patterns and disrupted small-world network properties. However, the organizational alterations in the autistic brain network are still poorly understood. We explored possible organizational alterations of 49 autistic children and 51 typically developing controls, by investigating their brain network metrics that are constructed upon cortical thickness correlations. Three modules were identified in controls, including cortical regions associated with brain functions of executive strategic, spatial/auditory/visual, and self-reference/episodic memory. There are also three modules found in autistic children with similar patterns. Compared with controls, autism demonstrates significantly reduced gross network modularity, and a larger number of inter-module connections. However, the autistic brain network demonstrates increased intra- and inter-module connectivity in brain regions including middle frontal gyrus, inferior parietal gyrus, and cingulate, suggesting one underlying compensatory mechanism associated with brain functions of self-reference and episodic memory. Results also show that there is increased correlation strength between regions inside frontal lobe, as well as impaired correlation strength between frontotemporal and frontoparietal regions. This alteration of correlation strength may contribute to the organization alteration of network structures in autistic brains.

## Introduction

Autism spectrum disorder is a complex developmental disability that characterized by deficits in social interaction, language skills, repetitive stereotyped behaviors and restricted interests. According to the latest estimate released in 2012 by the Centers for Disease Control and Prevention (CDC), Autism affects 1 in 88 American children, with a greater prevalence in boys, siblings of those with autism, and people with other developmental disorders such as Fragile X syndrome [1]. Autism usually starts before 3 years of age and lasts through the lifespan, although symptoms may improve over time. Autism is recently recognized as a heterogeneous disorder with multiple causes and courses, a great range in the severity of symptoms, and is associated with several co-morbid disorders [2].

A variety of neuroimaging studies have been performed in an attempt to understand the neurobiological underpinnings of autism [3]. For example, volumetric studies found gray matter and white matter abnormalities in frontal lobe [4], temporal lobe [5], [6], parietal lobe [7], and subcortical regions such as thalamus [8]. Moreover, cortical thickness was found to be thinner in left temporal and parietal regions in adolescents with autism [9]. Conversely, a study also reported a thicker cortex in the temporal and parietal lobes in younger autistic subjects with 8–12 years old [10]. Although great heterogeneity exists, the findings from structural aspects suggest that these volumetric abnormalities may contribute to atypical brain connectivity, and be responsible to their impaired ability of accomplishing complex cognitive and social tasks [11]. There is a hypothesis that autism is a neurodevelopmental disconnection syndrome, where disconnection in the autistic brain may be the result of a failure in the normal development of connectivity involving higher-order association areas, specifically, a combination of frontotemporal, frontolimbic, frontoparietal and interhemispheric connections [12]. It may also include weakening of already formed connections or a failure of certain connections in the correct initial organization [11].

Recent graph-based network analysis methods provide opportunities to investigate brain connectivity at a system level. The human brain can be considered to be a complex network where brain regions can be represented as nodes and inter-regional interactions as edges. Mathematical tools can be applied on the generated graphs to compute various network metrics that efficiently extract brain network properties [13]. The human brain network is generally comprised of several modules, e.g., community structures, which are more densely connected within modules than between the modules. This modularity property is ubiquitous in most complex systems in nature, ranging from social to biological networks [14]. A modular-organized network can evolve one module at a time while maintaining the functions of other modules, which is considered as a main advantage in evolutionary process and developmental optimization [15], [16]. Several recent studies have investigated various aspects of modular organization of large-scale brain networks in humans [17], [18]. Chen et al. built brain networks using cortical thickness correlations in 124 young adults with 24±4 years of age, and found that their networks are organized into 6 functionally oriented modules [19]. In their follow-up work, elderly adults with 76±9 years of age demonstrated less number of modules and significantly reduced modularity, when compared with young adults, which suggests an altered modular organization that may be induced by the reduced functional segregation in the aging brain [17]. Although the biological nature of such morphological networks remains unclear, the covary brain regions are suggested as a result of mutually trophic influences or common experience-related plasticity [20], [21]. There is a study demonstrating the pattern of cortical thickness correlation of certain brain regions (e.g., Brodmman area 44) is similar to the underlying fiber connections from DTI tractography [22]. Gong et al. also pointed out that approximately 35–40% convergent connections exist between brain networks using thickness and diffusion measurements, which suggest thickness correlations include exclusive information [23].

In this study, we propose to use modern modular organization techniques on the investigation of aberrant connectivity in autistic children. We hypothesize that the structural coupling between brain regions may be affected in autism, which eventually alters the modular organizations. A total of 49 autistic children and 51 typically developing controls were recruited at the age of 6–15 years. Brain networks were constructed for each population using inter-regional correlations of cortical thickness. Network metrics were finally computed from correlation maps, and statistically compared between autistic children and typically developing controls.

## Materials and Methods

### Subjects and MRI Acquisition

The participants in this study were derived from online public National Database for Autism Research (NDAR, http://ndar.nih.gov/) [24]. NDAR was created by the National Institutes of Health (NIH) as a repository for data sharing and scientific collaboration across autism investigators. Subjects were recruited across the United States with a population-based sampling method to assure that the samples recruited across all pediatric centers were demographically representative on the basis of variables that include age, gender, race, ethnicity, and socioeconomic status [25]. Informed consent from parents and children were obtained for all subjects.

This study includes 49 children with autism. Participants were included if they were between 6 and 15 years old, and their data contained standard autism assessments (e.g., the Autism Diagnostic Interview-Revised (ADI-R) and the Autism Diagnostic Observation Schedule (ADOS)). As a result, the current study included the following two collection IDs (along with submitters): NDARCOL0001356 (Bradley Peterson); and NDARCOL0001551 (Francisco Castellanos). The age and gender matched healthy subjects were selected from the Pediatric MRI Data Repository of NDAR, including 51 typically developing children (Table 1).

**Table 1:** Demographic and clinical details of participants.

Variables	Autism	Control
No. of subjects (n)	49	51
Gender (M/F)	40/9	39/12
Age (mean ± SD years)	9.6±2.2	9.7±2.1
Age range (years)	6–15	6–15
**Cognitive scores**		
ADI-R Social (mean ± SD)	17.6±7.6	–
ADI-R Communication (mean ± SD)	14.2±5.3	–
ADI-R Repetitive Behavior (mean ± SD)	4.9±2.6	–
ADOS Total (mean ± SD)	11.4±4.3	–

The subjects were scanned using 3T whole brain coverage MRI with 3D RF-spoiled gradient echo sequences. T1-weighted images were obtained, for sagittal slices in the resolution of 0.98×0.98×1 mm^3^ at the first autism collection, axial slices in the resolution of 1×1×1.33 mm^3^ at the second autism collection, and axial slices in the resolution of 1×1×1 mm^3^ at control subjects.

### Cortical Thickness Measurement

**Figure 1:**
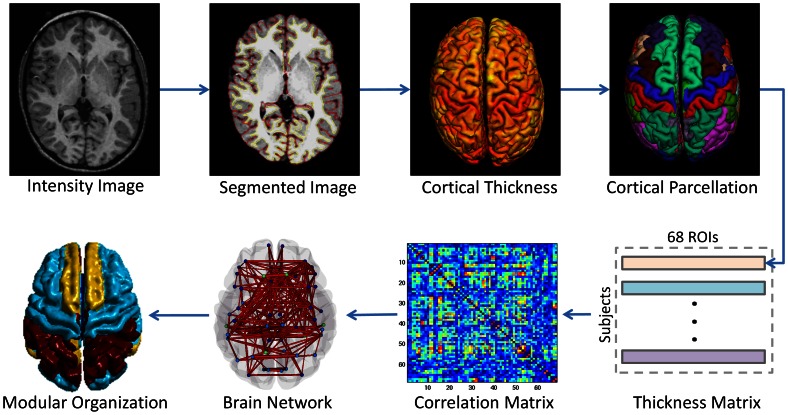
Flowchart for the construction of structural cortical network. For each subject, its original intensity image is first tissue-segmented, cortical-surface-constructed, thickness-measured, and surface parcellated into 68 ROIs. Then, it will serve as one entry of thickness matrix. A correlation matrix is finally obtained for each population group. The brain network is further built using ROIs as nodes and the correlations as edges, and modular organization is also computed.

Image processing and cortical reconstruction were performed with the Freesurfer image analysis suite (version 5.1.0), which is documented and freely available for download online (http://surfer.nmr.mgh.harvard.edu/). Recent studies have evaluated the performance of Freesurfer with images acquired from different sequences and scanners, and have concluded that reliable volumetric and surface measurements can be achieved although a minor bias may exist [26], [27]. As demonstrated in Fig. 1, all images were resampled into isotropic voxel of 1×1×1 mm^3^, intensity inhomogeneity corrected, and skull stripped. Cerebral white matter was segmented using a hybrid watershed classifier and the gray–white matter interface was identified. A deformable surface algorithm was then employed to search for the pial surface (the boundary between gray matter and CSF) using the gray–white matter interface as starting point. The whole cortex of each individual subject was visually inspected for segmentation inaccuracies and manually corrected if necessary. Local cortical thickness was measured as the distance between the inner and the outer cortical surfaces at each vertex [28].

By registering the Desikan-Killiany cortical atlas [29] to each subject, the brain was parcellated into 68 cortical regions (34 regions for each hemisphere) based on their anatomical patterns of gyrus and sulcus (Table 2).

**Table 2:** List of 68 anatomical regions comprising the cortical networks.

Index	Region	Abbreviation	Index	Region	Abbreviation
**1, 2**	Bank of the superior temporal sulcus	BSTS	35, 36	Pars orbitalis (Inferior frontal)	PORB
**3, 4**	Caudal anterior cingulate	CAC	37, 38	Pars triangularis (Inferior frontal)	PTRI
**5, 6**	Caudal middle frontal	CMF	39, 40	Pericalcarine	PERI
**7, 8**	Cuneus	CUN	41, 42	Postcentral gyrus	PSTC
**9, 10**	Entorhinal	ENT	43, 44	Posterior cingulate	PC
**11, 12**	Fusiform gyrus	FUSI	45, 46	Precentral gyrus	PREC
**13, 14**	Inferior parietal	IP	47, 48	Precuneus	PCUN
**15, 16**	Inferior temporal	IT	49, 50	Rostral anterior cingulate	RAC
**17, 18**	Isthmus of the cingulate	ISTC	51, 52	Rostral middle frontal	RMF
**19, 20**	Lateral occipital	LOCC	53, 54	Superior frontal	SF
**21, 22**	Lateral orbitofrontal	LOF	55, 56	Superior parietal	SP
**23, 24**	Lingual gyrus	LING	57, 58	Superior temporal	ST
**25, 26**	Medial orbitofrontal	MOF	59, 60	Supramarginal	SMAR
**27, 28**	Middle temporal	MT	61, 62	Frontal pole	FP
**29, 30**	Parahippocampal	PHG	63, 64	Temporal pole	TP
**31, 32**	Paracentral lobule	PARC	65, 66	Transverse temporal	TT
**33, 34**	Pars opercularis (Inferior frontal)	POPE	67, 68	Insula	INS

Odd numbers of index refer to the regions in left hemisphere, and even numbers of index refer to the regions in right hemisphere.

### Structural Brain Network Construction

For each population, the structural brain network was derived from a 68*68 correlation matrix computed from the regional cortical thickness of the 68 ROIs (Fig. 1). Briefly, mean cortical thickness was obtained for each ROI of each subject by averaging the vertices within the same anatomical label. Note that this measure is not affected by varied size of ROIs since it is an average per ROI. Before measuring the interregional correlations, a linear regression was performed at each ROI across all subjects to remove the effects of mean cortical thickness, age, gender, and age-gender interaction. The resulting residuals were used to replace the raw cortical thickness values. Pearson correlation was employed on the corrected cortical thickness for each pair of ROIs across all subjects in each population, and finally the structural networks *G* with *N* (68) nodes and *K* (2278, 68*67/2) weighted edges for both populations were obtained. For group comparison, edge weights in the networks of both the autism and healthy children were normalized by their total network weight, respectively [17].

### Network Modularity

A module of a complex network is a subset of nodes that are densely connected within the modules but sparsely connected between the modules. Modularity of a network is defined as [30]: 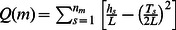, where *m* is the configuration of modular organization with 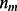 modules; *L* is the total weight of edges of this graph; 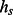 is the sum of the edge weights between nodes in module *s*; and 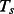 is the sum of the weights of nodes in module *s*, where the node weight is defined by the sum of edge weights connecting the node. An optimum network partition *m* is determined by assigning the nodes into a number of modules for achieving the maximum network modularity 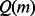. Note that the modules are non-overlapping and each node is assigned to one and only one module. Also, the process of modularity optimization does not need to specify the number, or the size of the modules.

We denote 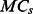 as the intra-module connectivity of module *s*, which is the sum of edge weights within module *s*. Similarly, the inter-module connectivity between module *s* and *t* is denoted as 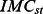, which is the sum of edge weights between the two modules. These two measures provide a module-level assessment of intra- and inter-module connectivity.

To further investigate the regional roles of cortical region, we define the intra-module degree (MD) and participation coefficient (PC) for each node as indices of their intra- and inter-module connection density, respectively [31]. For a node *i* in module *s*, its intra-module degree is measured by its regional intra-module connectivity 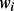, scaled by the average and standard deviation (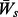 and 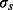) of the intra-modular degree for all nodes in the module: 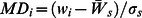. A high value of 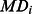 indicates strong within-module connectivity for node *i*. The participation coefficient, 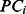, measures the regional inter-module connectivity of node *i*, and is defined as 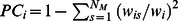, where 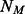 is the number of modules; 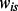 is the sum of edge weights between the node *i* and module *s*; 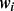 is the total weight of node *i* in the network. The PC of node *i* will be close to 0 if all weights are largely intra-modular.

More details of network measures can be found in [32]. We employ the Brain Connectivity Toolbox (http://www.brain-connectivity-toolbox.net) for the network measure computation.

### Statistical Analysis

To evaluate the significance of the network modularity obtained from real brain data, we compared it with the random networks generated with the same number of nodes and edges. Those random networks were created by randomly reassigning the edge weights within the same set of nodes, and the process was repeated for 10,000 times. The modularity difference between real network and random networks were assessed by one-sample t-test for significance evaluation.

To determine the statistical significance of measures between the autism and control networks, we utilized a non-parametric permutation test method [33]. First, we computed the difference of network measures between the constructed brain networks of the two groups. Then, we randomly reassigned the regional cortical thickness measures of each subject to either group, and recomputed the correlation matrix and network measures for each group. This randomization procedure was repeated 10,000 times, to determine the number of entries, *u,* where the between-group differences of permuted networks were larger or smaller than those of original networks. The significant level was thus calculated as *u* divided by the total number of permutations, i.e., 10,000.

To test whether interregional correlation of cortical thickness was significantly different between the autism and control networks, the correlation coefficients *r* were further converted into *z* values by using Fisher’s *r*-to-*z* transformation, 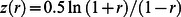 [34]. This transformation generated values that were approximately normally distributed. A *Z* statistic can be used to compare these transformed *z* values to determine the significance of the between-group correlation differences:
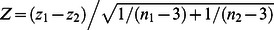



where 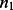 and 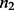 are the number of subjects of the two groups, and 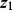 and 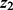 are the *z* values of the two groups at a pair of cortical regions, respectively. To adjust for the multiple comparisons, a false discovery rate (FDR) procedure was performed at a *q* value of 0.01 [35].

## Results

### Cortical Thickness

Cortical thickness maps were generated for each subject. Group mean of cortical thickness maps for autism and control groups can be visualized in Fig. S1, with the thickness ranging from 0 to 5 mm. In network construction, regional cortical thickness was calculated as an average across all vertices within a region. To illustrate the regional thickness variability, we picked two large regions, i.e., the superior frontal gyrus and precuneus, and ploted their mean and standard deviation of thickness in Fig. S2. Results demonstrate the standard deviation of thickness is consistently distributed between 0.5 to 1 mm. We further compared ROI-based thickness differences between autism and control groups, and found significant lower thickness in autism subjects in ROIs of bilateral middle frontal gyrus, right superior frontal gyrus, and bilateral temporal pole (Fig. S3). Autism subjects also demonstrate higher thickness in bilateral paracentral lobe. Regression revealed a negative linear correlation between thickness and age for both groups, agreeing with previous findings [36].

### Modular Organizations of Autistic Brain

**Figure 2:**
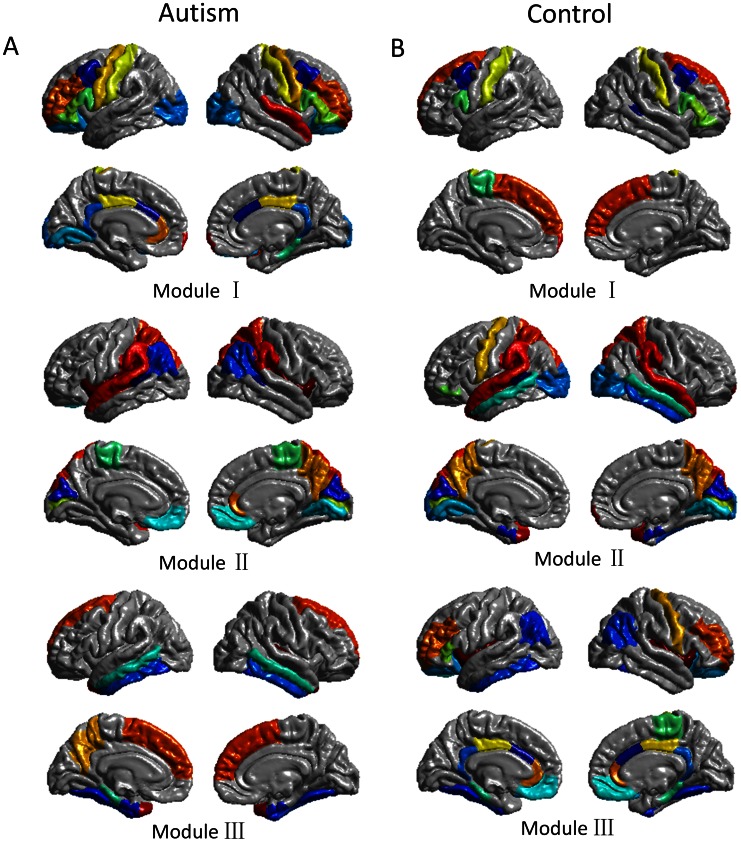
Modular organization of the cortical structural network of autistic children and typically developing children. (A) The brain modular organization of autistic children. Module I: executive strategic, sensorimotor, and visual; Module II: spatial and auditory; Module III: recognition and self-awareness. (B) The brain modular organization of typically developing children. Module I: executive strategic; Module II: spatial, auditory, and visual; Module III: self-reference and episodic memory. Colors in each module represent different cortical regions.

Three functionally oriented modules were discovered in the autistic brain, as shown in Fig. 2A. Module I includes 30 regions including bilateral middle frontal gyrus, precentral gyrus (PREC), postcentral gyrus (PSTC), and lateral occipital gyrus (LOCC), which corresponds to executive, sensorimotor, and visual functions. A total of 23 regions are included in module II, consisting of superior parietal gyrus (SP), inferior parietal gyrus (IP), supramarginal gyrus (SMAR), and superior temporal gyrus (ST), mainly associated with spatial and auditory functions. The third module includes 15 brain regions from superior frontal gyrus (SF), inferior and middle temporal gyrus (IT, MT), and precuneus gyrus (PCUN), which are related with recognition and self-awareness functions.

### Modular Organizations of Typically Developing Brain

We detected 3 modules in the typically developing controls, as shown in Fig. 2B. Module I has 13 brain regions, including SF, caudal middle frontal gyrus (CMF), and PSTC, associated with executive strategic functions. Module II contains 28 brain regions, such as SP, SMAR, ST, MT, and LOCC, which are known primarily involved in spatial, auditory, and visual functions. Module III consists of 27 regions including rostral middle frontal gyrus (RMF), medial orbitofrontal gyrus (MOF), rostral anterior and posterior cingulate (RAC and PC), and inferior parietal gyrus (IP), which are mainly related to brain functions of self-reference and episodic memory.

### Brain Modularity: Autism vs. Control

#### Global brain modularity

We determined the brain module patterns that achieved maximum modularity in the weighted brain networks of autism and healthy controls, respectively. Specifically, three modules were found in the networks of both populations. Non-random modularity was confirmed, as both networks exhibit significant higher modularity than those of 10,000 random networks (*p*<0.001).

**Figure 3:**
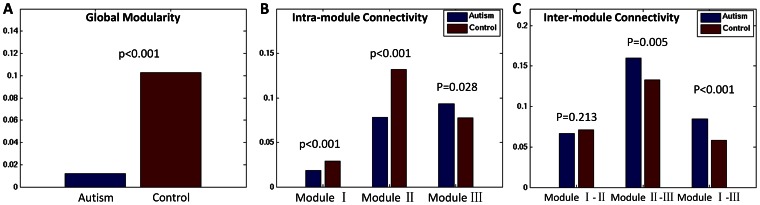
Altered network modularity. (A) Between-group difference in the network modularity. The network modularity of the autism (left) is significantly reduced when compared with the control (right). (B) Between-group difference in the intra-module connectivity (MC). Autism showed a significantly reduced connectivity in the modules I and II, while demonstrated a significantly increased connectivity in the module III. (C) Between-group differences in the inter-module connectivity (IMC). Autism showed a significantly increased connectivity between modules II and III, and between modules I and III. No significant difference was found for the connectivity between modules I and II.

We consider the typically developing control subjects have ‘ideal’ modular organization for the brain network, which is a more optimized design than that of autism subjects [17]. Therefore, we take the modular organization in control subjects as baseline, and the comparisons between the autism and control networks are focused on the same modular organization (3 modules, Fig. 2B) derived from the control network. This strategy has been proposed in a previous study that used young adults as baseline for analyzing the modular organization alterations in aging subjects [17]. In this study, we recalculated the modularity of autism network with the modular organization derived from control network. Not surprisingly, a significant lower modularity (*p*<0.001) was found in autism network (Fig. 3A).

#### Alterations in module connectivity

We then examined the intra-module connectivity in both populations using the modular organization from the controls (Fig. 3B). Autism subjects demonstrated a significant decrease in intra-module connectivity for both modules I and II, indicating fewer connections inside the brain regions with functions of executive control and spatial/auditory/visual processing, respectively. Surprisingly, autism subjects showed significantly higher connectivity in the brain regions with functions of self-reference and episodic memory (module III, *p* = 0.028).

Inter-module connectivity measures the connections in between the 3 modules derived from the controls (Fig. 3C). A significantly larger number of inter-module connections was found in autistic brain network such as modules II-III, and I-III. This is expected since autism has previously demonstrated lower overall modularity and thus has more inter-module connections. No significant difference was found in modules I-II.

#### Regional roles of alterations in module connectivity

**Figure 4:**
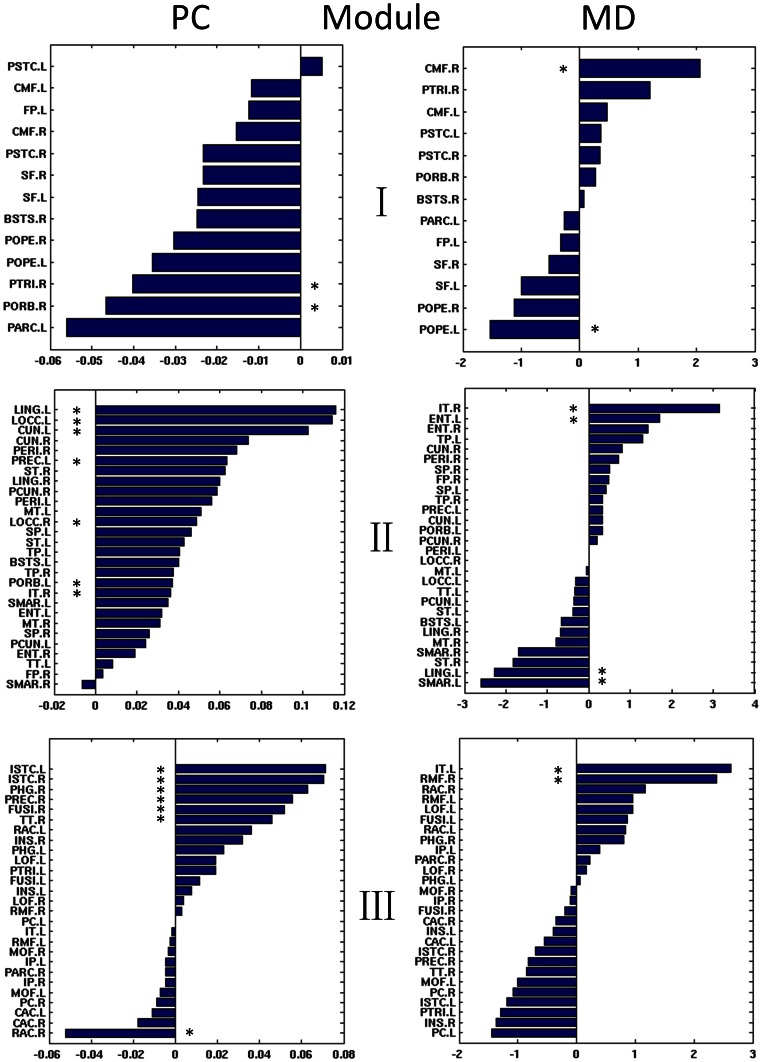
Regional roles of network modularity. The between-group comparisons identified 16 and 9 regions with significant altered Participant Coefficient (PC) and intra-module degree (MD) values in the autistic brain, respectively. Positive values mean that autism has larger connectivity than healthy children. Stars indicate the regions with significant difference in the number of connections between groups in 10,000 permutation tests (p<0.01, FDR corrected).

We further explore the disease-related alterations in the regional roles of the intra- and inter-module networks (Fig. 4). Similarly, modules are defined using the control network. Specifically, the Participant Coefficient was used to measure the regional inter-module connectivity of each brain region. As a result, sixteen regions were identified significantly altered, in which a majority of regions (13 out of 16) have increased PC in the autism network than in the control network. This is consistent with the previous finding that autism has larger overall inter-module connectivity. The regions showing increased PC are mostly located in the frontal (e.g., PREC), right temporal (e.g., ST, IT), occipital (e.g., LOCC, CUN.L, LING.L), and limbic lobes (e.g., ISTC, PHG.R, FUSI.R, and TT.R). Regional intra-module degree of each node was also examined, and 8 regions were found to be significantly altered. Interestingly, 5 out of 8 regions show increased MD in medial frontal regions (CMF.R, RMF.R) and temporal lobe (IT, ENT.L). Decreased MD was found in the inferior frontal (POPE.L), parietal (SMAR.L), and occipital lobes (LING.L).

### Altered Inter-regional correlations in the Autism Network

**Figure 5:**
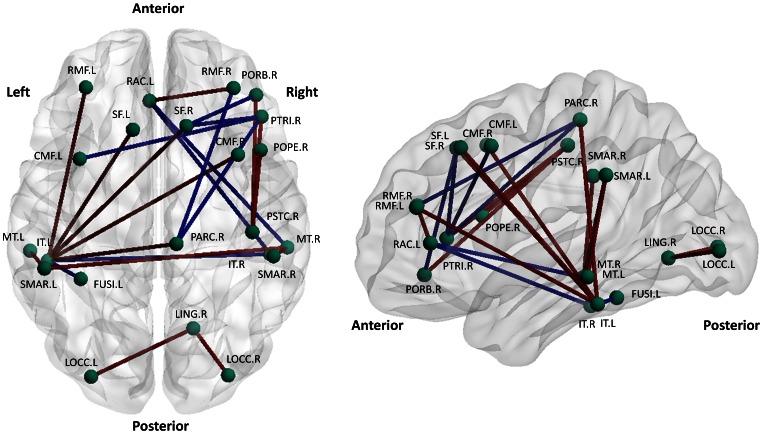
Illustration of connections with significantly altered regional correlations in the autism network. Left shows the top view, and right shows the lateral view. Decreased correlations in autism were marked in red, and increased correlations were marked in blue. Visualization was implemented using BrainNet Viewer (http://www.nitrc.org/projects/bnv/).

The inter-regional correlation strength was also examined (Table 3). We identified 14 decreased correlations and 9 increased correlations in autism, when compared with control (p<0.01, FDR corrected). As visualized in Fig. 5, the decreased connections are distributed at the frontal lobes, temporal-parietal lobes, and inside the occipital lobes. Specifically, the frontal regions have decreased correlations with regions in parietal, temporal, and limbic lobes. The left inferior temporal gyrus (IT.L) establishes decreased correlations with bilateral CMF, paracentral gyrus (PARC), PSTC, and ST. Also, connections between MT and SMAR were decreased. Increased connections were also found, including regions inside frontal lobe, between frontal and paracentral regions, and between frontal and temporal regions such as fusiform (FUSI) and IT.

**Table 3:** Altered regional correlations in autistic and control networks (p<0.01 FDR corrected).

Region A	Region B	Regional correlation *r*		*Z* score	Module	Note
		Autism	Control			
**Decreased correlations in autism**						
POPE.R	PSTC.R	−0.45	0.43	**4.61**	I	Frontal-parietal
PORB.R	PSTC.R	−0.37	0.47	**4.37**	I	
PTRI.R	PSTC.R	−0.48	0.35	**4.28**	I	
IT.L	CMF.R	−0.62	0.17	**4.38**	I–III	Frontal-temporal
IT.L	SF.L	−0.59	0.21	**4.33**	I–III	
IT.L	SF.R	−0.76	0.08	**5.21**	I–III	
IT.L	PARC.R	−0.59	0.22	**4.36**	III	
IT.L	RMF.L	−0.63	0.1	**4.06**	III	
RAC.L	RMF.R	−0.62	0.26	**4.81**	III	Frontal-limbic
MT.L	SMAR.L	−0.02	0.71	**4.38**	II	Temporal-parietal
MT.R	SMAR.L	−0.17	0.61	**4.3**	II	
MT.R	SMAR.R	−0.3	0.61	**4.99**	II	
LOCC.L	LING.R	−0.26	0.55	**4.27**	II	Occipital
LOCC.R	LING.R	−0.25	0.56	**4.33**	II	
**Increased correlations in autism**						
CMF.L	PTRI.R	0.45	−0.51	−**5.05**	I	Frontal
PARC.R	RMF.R	0.63	−0.53	−**6.48**	III	
PORB.R	SF.R	0.51	−0.34	−**4.46**	I	
PTRI.R	SF.R	0.61	−0.32	−**5.07**	I	
PARC.R	PTRI.R	0.54	−0.32	−**4.52**	I–III	
IT.L	FUSI.L	0.81	0.28	−**4.11**	III	Temporal
IT.L	IT.R	0.8	0	−**5.27**	II–III	
IT.R	RAC.L	0.6	−0.26	−**4.66**	II–III	Temporal-limbic
MT.R	RAC.L	0.52	−0.3	−**4.34**	II–III	

## Discussion

In this study, we performed a modularity analysis of structural cortical networks of autistic children in comparison to typically developing control children. Anatomically meaningful cortical regions were used as nodes and the correlations of inter-regional cortical thickness were taken as connections. Graph-based theory was applied on the fully weighted networks, to avoid the limitation of threshold selection in the analysis of binarized networks [37]. Our main findings are as follows: 1) when compared to healthy children, autistic children presented a significantly reduced modularity, which may result from a re-organization of the brain network; 2) increased intra- and inter-module connectivity were both found among the module associated with brain functions of self-reference and episodic memory in autism; 3) inter-regional correlations revealed increased intra-connections in the frontal lobe, and decreased connections between frontal lobe and other lobes such as temporal and parietal, which may contribute to the alteration of modular organization.

### Reduced Modularity in Autistic Brain

We found that both autism and control children demonstrate a highly non-random modularity. Many previous studies have proven that biomedical networks, such as human brain networks, have small-world architecture [38]. Further analysis reveals that the network can be efficiently represented as several communities, i.e., modular organization. It has been suggested that such modular organization provides a balance between brain functional segregation and integration, which are the two most fundamental aspects of brain organization [39].

However, when we apply the modular organization of typically developing controls to autism children, the network of the latter demonstrates a significantly reduced modularity (*p*<0.001) (Fig. 3A). This suggests that the modular organization in controls is not optimal for autism. Instead, the autistic children may have re-organized their brain networks in part to offset the cortical thickness alterations (Fig. S1), inter-regional correlation abnormalities, and/or maintain cognitive functions and daily living activities [3]. This re-organization could be a result of the autistic brain following a different developmental trajectory. Raznahan et al. found that cortical thickness was smaller in autism in childhood but progressively thickened in adulthood [40]. Studies have reported that brain networks in individuals with Autism have altered small-world network properties and different patterns of connectivity in an EEG study [41]. Moreover, network re-organization has been proposed in many other studies. For example, van den Heuvel et al. reported that the global organization of the brain network was affected in schizophrenia patients, although local connectivity was found intact [42]. Chen et al. reported that the altered modular organization in aging population may be associated with their declined cognitive functions [17], [43].

### Altered Modular Organization in Autistic Brain

We identified 3 functionally oriented modules in typically developing controls. Particular interest was focused on module III, in which the brain regions are associated with brain functions of self-reference and episodic memory. Note that those regions are related with default mode network [44]. As observed from Fig. 3B and 3C, autistic children have surprisingly higher intra-module connectivity in this module (*p* = 0.028), as well as larger inter-module connectivity to the other two modules such as I-III (*p*<0.001) and II-III (*p* = 0.005). Regional role analysis found significant higher inter-module connectivity in the autistic brain within the cortical regions of limbic (ISTC, PHG), frontal (PREC.R), and temporal lobes (FUSI.R, TT.R), and higher intra-module connectivity between the cortical regions of frontal (RMF.R) and temporal lobes (IT.L) (Fig. 4). Recalling that we have normalized the total edge strength of autism cortical network to be equal with that of control network (see Method section for details), the findings suggest that the autism network organized higher edge strength inside module III for the self-related function, as well as in between module III and other two modules. Autism literally means “self”, derived from Greek word “autos” [45]. This finding confirms with behavioral observations that autism exhibits self-focus characteristic [46].

This finding only suggests a weighting distribution alteration in the autism network, therefore it cannot be implied that the functioning of regions in module III is more efficient in autism than in control. Instead, functional MRI studies have proposed that individuals with autism have broad impairments in self-referential cognition, empathy [47], and diminished episodic memory and episodic future thinking [48]. Our results may demonstrate that individuals with autism employ a compensatory strategy that relies more on self-related functions to remedy their functional deficits. Previous study also reported that individuals with autism may employ compensatory strategies to carry out the self-report task, due to the impairment in the expression of conscious feelings [49].

We also found decreased intra-module connectivity in autism in executive strategic module (Fig. 3B). Regional role analysis reveals that the inferior frontal gyrus has significant less inter-module connections (PTRI.R and PORB.R) and intra-module connections (POPE.L). Deficits in executive functioning are one of the core criteria for the diagnosis of autism. Individuals with autism experience difficulty in processing large amounts of information and relating to others, including skills such as organizing, planning, sustaining attention, and inhibiting inappropriate responses [50]. For example, Corbett et al. found significant deficits of executive functioning in autistic children compared to controls, especially for vigilance, response inhibition, cognitive flexibility/switching, and working memory. We found less connectivity in the module related with executive functioning, which may contribute to the behavior difficulties in autism, such as paying attention to big picture, complex thinking, and organizing their thoughts and actions.

From the brain development point of view, it has been proposed that the early human brain prefers to integrate brain functions from regions that are anatomically close before favoring connections between further apart, otherwise known as local-to-distributed development [51]. As the progress of brain maturation and myelination, long-distance connections start to establish, which leads to a global reduction in shortest path between components, as called economical small-world properties [39]. The subjects in our study, aged at the 6–15 years, presented 3 distinct modules, which is larger than the 2 modules reported in infants [52] and smaller than the 5–6 modules found in young adults (Chen et al., 2008, Chen et al., 2011).

### Altered Inter-regional Correlations in the Autism Network

The alteration of inter-regional correlations may be one of the major reasons re-organizing the module distribution. We have 3 major findings. First, 5 out of 9 connections with stronger correlation strength are located in frontal lobe (Table 3, Fig. 5). This suggests that frontal lobe may have excessive local connections and is in agreement with previous studies. For example, Courchesne et al. proposed that the frontal cortex in autism might tend to talk with itself but failed to hear and respond to other brain systems due to local over-connectivity [53]. Second, the connection strengths between frontal lobe and parietal, temporal, and limbic lobes are significantly reduced (Table 3). This may affect the functional integration between frontal regions with other regions. Barttfeld et al. performed a EEG study on dynamic brain connectivity and found that individuals with autism exhibited an excess of short-range connections and a deficit in long-range connections, which is consistent with findings in [41]. Previous studies have also reported the decreased and/or impaired frontal-parietal connection in autism, which may affect top-down processing [54]. Third, regions in temporal lobe (e.g., MT) have significantly reduced connection strength with regions in parietal lobe (e.g., SMAR). Note that MT is considered playing a causal role in executive dysfunction in autism [55]. Therefore, the deficits of the temporo-parietal connection may contribute to the impairment of the executive function in autism [56].

We found medial frontal regions (CMF and RMF) have increased connections with frontal lobe but decreased connections with other lobes such as temporal and limbic lobes. Those two regions serve as major functional components in the theory of mind (ToM), which refers the ability to attribute mental states to oneself and others. The findings support the notion that the functional integration across different regions in ToM is impaired in autistic children [11]. Meanwhile, we found that IT has increased correlation with temporal region but decreased correlation with frontal regions, which is reported as an active component in ToM [57]. However, as another component in ToM, no significant correlation strength was found in ST.

### Methodological Issues

There are also some methodological issues in this study. First, we employed the brain parcellation scheme using the Desikan-Killiany cortical atlas, which includes 68 cortical regions. As Zalesky et al. pointed out, network parameters vary considerably as a function of spatial scale [58]. That is, it would only be meaningful when the findings are compared with the studies having similar number of network nodes. Second, the regional interactions studied here are only for cortical regions. Subcortical regions may be also interesting, and has been reported involving with the reduced ability of information processing in autism [59]. Third, our data has an uneven gender distribution with more boys than girls, although we did our best to remove the gender and age effect by using the regression model. The gender effect analysis is not carried out due to the small sample size of girls, which may be addressed in future studies as NDAR repositories are including more subjects.

### Conclusion

In conclusion, we conducted a module-based brain network analysis in autistic children and typically developing controls using inter-regional cortical thickness correlations. We observed a significant reduced modularity in autism when compared to controls. Increased intra- and inter-module connectivity were found in the modules associated with brain functions of self-reference and episodic memory, suggesting the underlying compensatory mechanism in autistic brain. Increased correlation strength was found within frontal regions while long distance connections between frontal and other lobes such as temporal, parietal, and limbic lobes experienced reduced correlation strength. In general, the results confirmed our hypothesis that autism has altered inter-regional correlations, which further contributes to the alteration of modular organization. The present study provides insightful implications on the understanding of how the brain regions interact atypically in children with autism.

## Supporting Information

Figure S1 Mean cortical thickness in both autism and control groups.
(TIF)

Figure S2 Illustration of regional cortical thickness variability in superior frontal gyrus and precuneus. Mean and standard deviation of thickness in each subject were plotted.
(TIF)

Figure S3 Region-based cortical thickness comparisons between autism and control groups. Autism subjects have significant lower cortical thickness in the blue regions while higher cortical thickness in the red regions. Bottom shows the cortical thickness as a function of age for these significant regions.
(TIF)
