# Supplementary figures and images for: Altered Modular Organization of Structural Cortical Networks in Children with Autism
Source: PLoS One. 2013 May 10;8(5):e63131. doi: 10.1371/journal.pone.0063131 (PMC3651174; doi:10.1371/journal.pone.0063131)

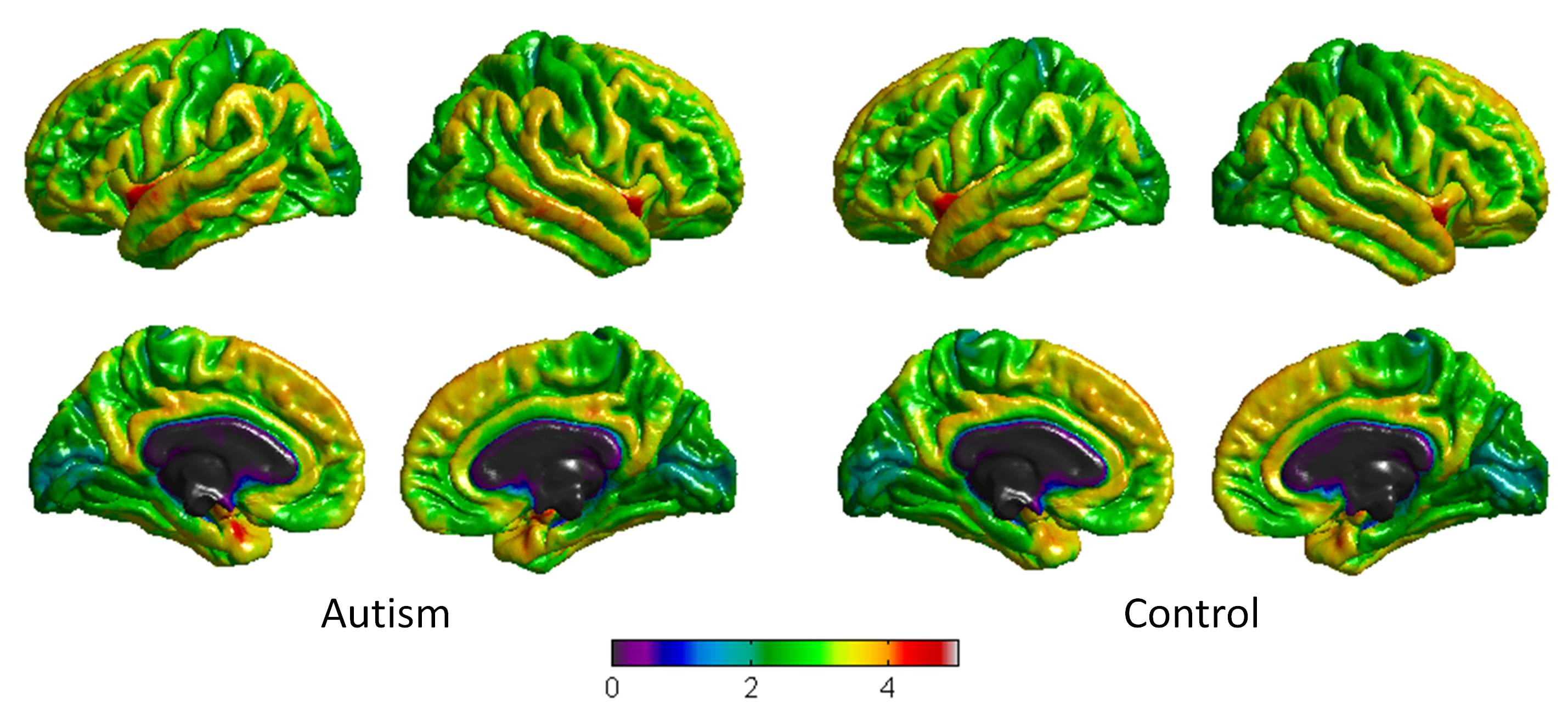

Supplement: Figure S1 — Mean cortical thickness in both autism and control groups. (TIF) [file pone.0063131.s001.tif]

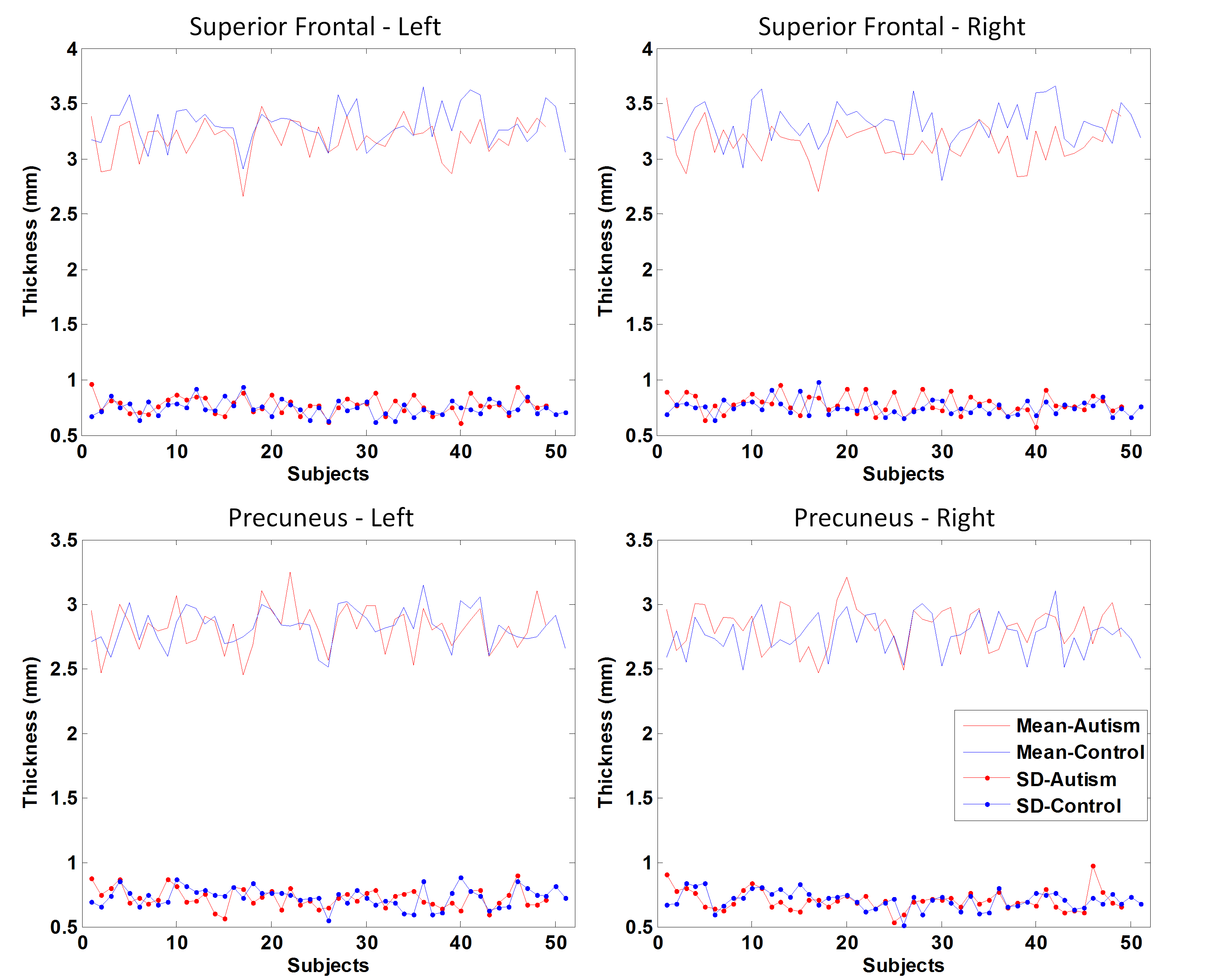

Supplement: Figure S2 — Illustration of regional cortical thickness variability in superior frontal gyrus and precuneus. Mean and standard deviation of thickness in each subject were plotted. (TIF) [file pone.0063131.s002.tif]

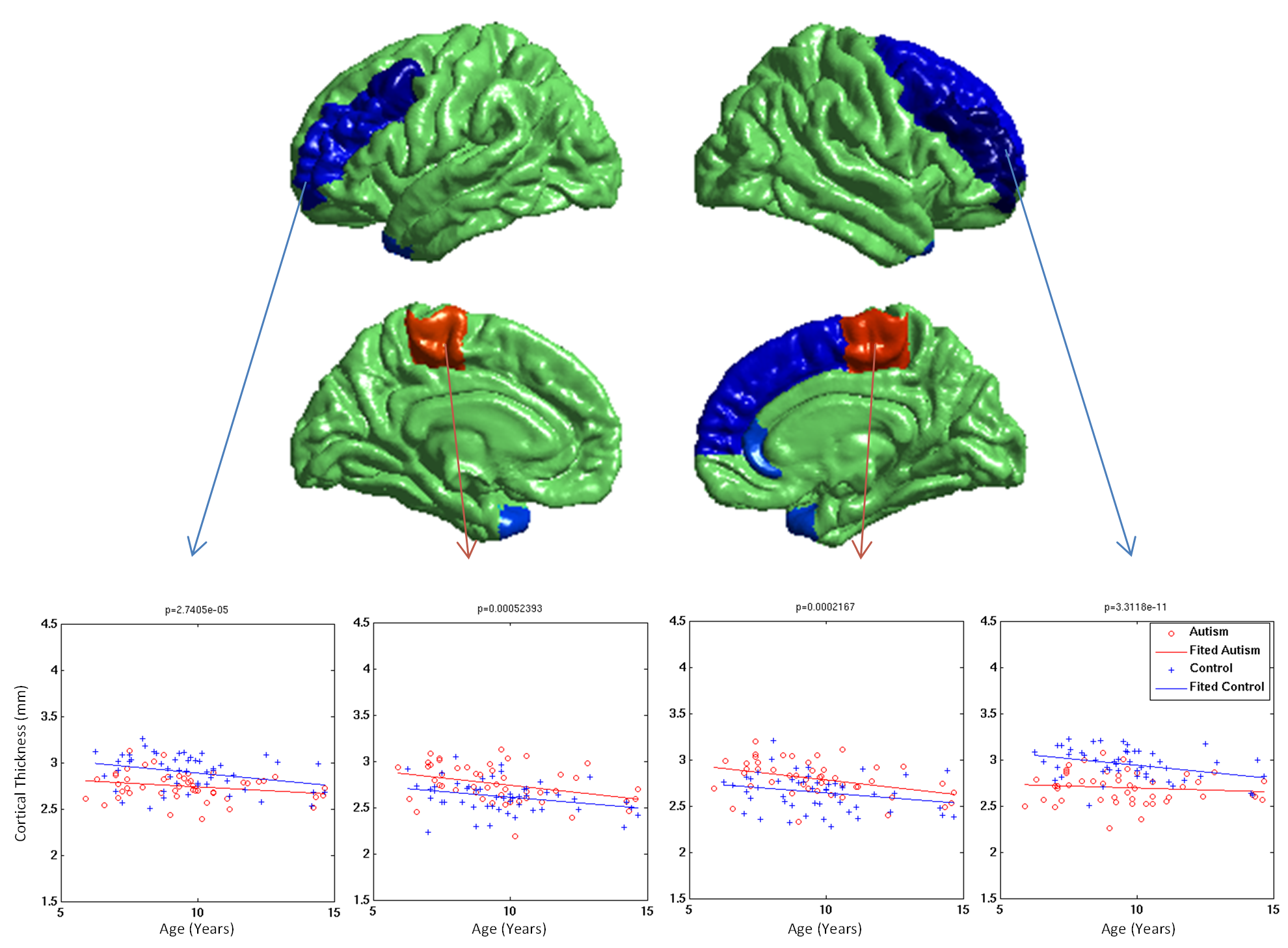

Supplement: Figure S3 — Region-based cortical thickness comparisons between autism and control groups. Autism subjects have significant lower cortical thickness in the blue regions while higher cortical thickness in the red regions. Bottom shows the cortical thickness as a function of age for these significant regions. (TIF) [file pone.0063131.s003.tif]
